# Anxiolytic effects of Chrysanthemum morifolium Ramat Carbonisata-based carbon dots in mCPP-induced anxiety-like behavior in mice: a nature-inspired approach

**DOI:** 10.3389/fmolb.2023.1222415

**Published:** 2023-07-13

**Authors:** Luming Cui, Qian Zhang, Yifan Zhang, Tingjie Li, Menghan Li, Jinye Yuan, Zhiyi Wu, Yue Zhang, Hui Kong, Huihua Qu, Yan Zhao

**Affiliations:** ^1^ School of Traditional Chinese Medicine, Beijing University of Chinese Medicine, Beijing, China; ^2^ Qingdao Zhonghengneng Environmental Science Engineering Research Institute Co., Ltd, Qingdao, China; ^3^ School of Chinese Materia Medica, Beijing University of Chinese Medicine, Beijing, China; ^4^ School of Life Sciences, Beijing University of Chinese Medicine, Beijing, China; ^5^ Center of Scientific Experiment, Beijing University of Chinese Medicine, Beijing, China

**Keywords:** carbon dots, Chrysanthemum morifolium Ramat, anxiety disorder, HPA axis, neurotransmitters

## Abstract

**Introduction:** Anxiety disorders have emerged as a predominant health concern, yet existing pharmacological treatments for anxiety still present various challenges. Chrysanthemum morifolium Ramat Carbonisata (CMRC) has been utilized in China for approximately 400 years as a therapeutic intervention for anxiety disorders. In this study, a novel type of carbon dots derived from the decoction of Chrysanthemum morifolium Ramat Carbonisata (CMRC-CDs) was identified and isolated, and their morphological structure and functional groups were characterized. Furthermore, the effects of CMRC-CDs on m-chlorophenylpiperazine (mCPP)-induced anxiety-like behaviour in mice were examined and quantified. In order to investigate the potential mechanisms of their anxiolytic effects, concentrations of hypothalamic-pituitary-adrenal (HPA) axis hormones, amino acid neurotransmitters, and monoamine neurotransmitters were measured.

**Methods:** In this study, we synthesized CMRC-CDs and evaluated their potential anti-anxiety effects in a controlled experiment involving 48 male ICR mice. The mice were randomly divided into six groups, treated with CMRC-CDs at different doses for 14 days, and subjected to Open-Field (OF) and Elevated Plus Maze (EPM) tests. Post-behavioral evaluations, blood samples and brain tissues were collected for neurotransmitter and Hypothalamic-Pituitary-Adrenal (HPA) axis hormone quantification via ELISA. Additionally, cytotoxicity of CMRC-CDs was assessed using a Cell Counting Kit-8 (CCK-8) assay on RAW 264.7 cells.

**Results and Discussion:** CMRC-CDs were spherical and homogeneously dispersed, with diameters ranging from 1.4 to 4.0 nm and an abundance of chemical groups on their surface. In the open-field (OF) test, mice pre-treated with CMRC-CDs demonstrated an increased proportion of time spent in the central area and a higher frequency of entries into the central area. In the elevated plus maze (EPM) test, mice pre-treated with CMRC-CDs exhibited a greater number of entries into the open arm and an extended duration spent in the open arm. CMRC-CDs were observed to decrease serum concentrations of corticotropin-releasing hormone (CRH), adrenocorticotropic hormone (ACTH), and corticosterone (CORT). Furthermore, CMRC-CDs were found to increase γ-aminobutyric acid (GABA) and 5-hydroxytryptamine (5-HT) levels, while concurrently reducing glutamic acid (Glu) concentrations in brain tissue. CMRC-CDs demonstrated anxiolytic effects, which may be attributed to their modulation of hormones and neurotransmitters. This finding suggests the potential therapeutic value of CMRC-CDs in the clinical treatment of anxiety disorders.

## 1 Introduction

The prevalence of mental disorders has steadily increased since 1990. By 2019, anxiety disorders had emerged as one of the leading causes of global burden, ranking 24th among the primary causes of disability-adjusted life years (“Global, Regional, and National Burden of 12 Mental Disorders in 204 Countries and Territories, 1990–2019: A Systematic Analysis for the Global Burden of Disease Study, 2019”^2022^). In recent years, the COVID-19 pandemic has further escalated the prevalence of anxiety disorders (Santomauro et al., 2021). According to one study estimate, one-third of adults experienced anxiety during the global coronavirus disease outbreak in 2019 (Delpino et al., 2022). Consequently, the treatment of anxiety disorders is garnering increased attention.

The pathophysiology of anxiety disorders remains an area ripe for further exploration, but it is generally believed to be closely associated with the hypothalamic-pituitary-adrenocortical (HPA) axis and neurotransmitter secretion (Meldrum, 2000; Jacobson, 2014; Olivier and Olivier, 2020). Currently, prevalent anti-anxiety medications include benzodiazepines, selective serotonin reuptake inhibitors (SSRIs), and serotonin-noradrenaline reuptake inhibitors (SNRIs). Nevertheless, these drugs present several challenges, such as benzodiazepines causing side effects like increased talkativeness, emotional release, excitement, and excessive movement; SSRIs leading to sexual dysfunction; and both causing potent withdrawal symptoms (Petursson, 1994; Mancuso et al., 2004; Bala et al., 2018; Horowitz and Taylor, 2019). These issues have prompted a search for and investigation into more effective and safer anti-anxiety drugs.

Carbon dots (CDs), featuring ultra-fine dimensions of below 10 nm, were first identified in 2004 (Cui et al., 2021). Due to their minimal cytotoxicity, superior biocompatibility, chemical stability, negligible toxicity, and substantial surface area-to-volume ratio, they are finding escalating usage in the realm of biomedical applications (Durán et al., 2016; Jaleel and Pramod, 2018; Singh et al., 2018; Ross et al., 2020; Khayal et al., 2021; Mansuriya and Altintas, 2021). A myriad of studies have underscored the potential of carbon dots, suggesting their prospective utility as innovative carriers for drug delivery systems targeting the central nervous system. These studies further posit that carbon dots may serve as a therapeutic intervention for an array of psychiatric and cognitive disorders (Ashrafizadeh et al., 2020; Henna et al., 2020). For instance, nanodiamonds have been observed to exhibit neuroprotective effects against Alzheimer’s disease (Alawdi et al., 2017), graphene oxide has been shown to mitigate neurotoxicity and improve cognitive impairment (Ren et al., 2018; Chu et al., 2021), and graphene quantum dots have been found to enhance learning abilities (Xiao et al., 2016).

In recent years, the number of raw material options for synthesizing CDs has expanded significantly, and the extraction of CDs from various natural sources, especially plants, is attracting increasing attention due to its convenience and affordability. As a result, numerous studies have emerged on the biomedical applications of carbon dots derived from herbal medicines, particularly carbonized traditional herbs (Chen et al., 2019; Luo et al., 2021; Li et al., 2022). A variety of herbs used in traditional Chinese medicine to treat mental symptoms have been investigated for their active ingredients and mechanisms of action, such as Rhodiola rosea, ginseng, and Ginkgo biloba (S. Lee and Rhee, 2017; Liu et al., 2015; Xie et al., 2018; Panossian et al., 2010). However, the herbs that have been evaluated so far represent only a small proportion of those in daily use, suggesting that the effects and mechanisms of single herbs in improving mood disorders still warrant further research.

Given this background, we embarked on a study aimed at exploring the anxiolytic effects and underlying mechanisms of carbonized derivatives sourced from a commonly used and widely available traditional herb. Chrysanthemum morifolium Ramat (CMR), known as “Jv Hua” in Chinese, boasts a longstanding medicinal tradition within China. Chrysanthemum morifolium Ramat Carbonisata (CMRC) is a CMR product obtained through the carbonization process. It has been used as a sedative in China since its first documentation in the “Guide to Clinical Practice with Medical Records” over 300 years ago. Nevertheless, its efficacy and underlying mechanisms remain incompletely understood, highlighting the need for further investigation.

In this study, we synthesized CMRC-based carbon dots (CMRC-CDs) using an eco-friendly approach and evaluated their physicochemical properties, such as morphology and functional groups. We utilized a variety of analytical techniques, encompassing roadmaps, heat maps, and regional dwell times, to yield a thorough evaluation of the behavioral outcomes. These metrics were utilized to determine the capacity of CMRC-CDs to alleviate anxiety-like behavior induced by mCPP administration in mice. The underlying mechanisms of the anxiolytic effects were explored by measuring the levels of corticotropin-releasing hormone (CRH), adrenocorticotropic hormone (ACTH), corticosterone (CORT), 5-hydroxytryptamine (5-HT), dopamine (DA), norepinephrine (NE), glutamic acid (Glu), and γ-aminobutyric acid (GABA). Our findings may provide a valuable contribution towards comprehending the anxiolytic properties of CMRC-CDs and offer valuable insights into the potential utility of carbon dots obtained from traditional herbs for the development of novel, efficacious, and safer anti-anxiety therapies.

## 2 Results

### 2.1 Analysis of the properties and features of CMRC-CDs

**FIGURE 1:**
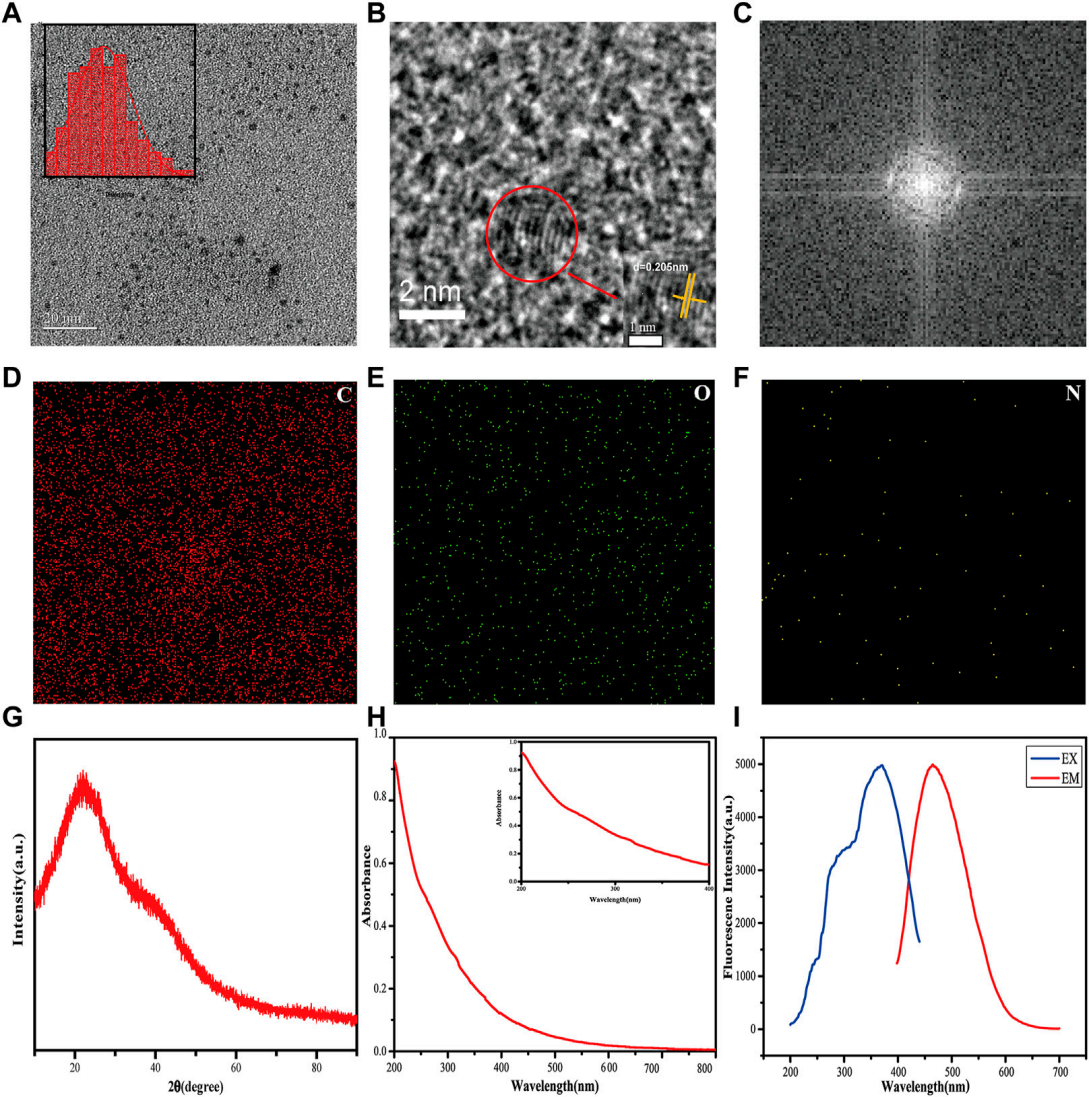
Characterization of CMRC-CDs. **(A)** Transmission electron microscopy (TEM) image of CMRC-CDs, with histogram depicting particle size distribution of CMRC-CDs. **(B)** High-resolution TEM (HRTEM) image of individual CMRC-CDs **(C)** FFT image processing was performed on CMCR-CDs. **(D–F)** TEM mapping of CMRC-CDs, showing the main elements in carbon dots, including C, O, and N. **(G)** XRD pattern spectrum of CMRC-CDs. **(H)** UV-vis absorption spectrum of CMRC-CDs. **(I)** Fluorescence excitation spectra and emission spectra of CMRC-CDs.

As depicted in Figure 1A, TEM micrographs reveal that the CMRC-CDs exhibit a nearly spherical morphology and are uniformly dispersed throughout the sample. The diameters of the CMRC-CDs span between 1.4 and 4.0 nm, with a majority of particles concentrated within the 1.8–2.8 nm interval. Furthermore, HRTEM imaging demonstrates a lattice spacing of 0.205 nm for the CMRC-CDs, as illustrated in Figure 1B. Figure 1C presents the results obtained from the Fast Fourier Transform (FFT) analysis performed on CMRC-CDs, facilitating a clearer visualization of the lattice structure.

Figures 1D–F depict TEM elemental mappings of the CMRC-CDs, illustrating the primary constituents within the carbon dots as carbon (C), oxygen (O), and nitrogen (N), while concurrently displaying their spatial distribution.

Figure 1G illustrates a distinct diffraction peak at 2θ = 22.0° in the X-ray diffraction (XRD) pattern of CMRC-CDs. When considered in conjunction with the High-Resolution Transmission Electron Microscopy (HRTEM) imaging results, it becomes apparent that CMRC-CDs represent a carbon structure that resides between amorphous and lattice morphologies.

In the aqueous solution, the UV-Vis absorption spectrum of the CMRC-CDs exhibited a subtle absorption peak at 310 nm, which is indicative of the π–π* electronic transitions of the aromatic C=C and C≡C bonds (Figure 1H) (Y. Zhang et al., 2021). Furthermore, the fluorescence characterization of the CMRC-CDs revealed a maximum emission at 465 nm upon excitation at a wavelength of 369 nm (Figure 1I).

**FIGURE 2:**
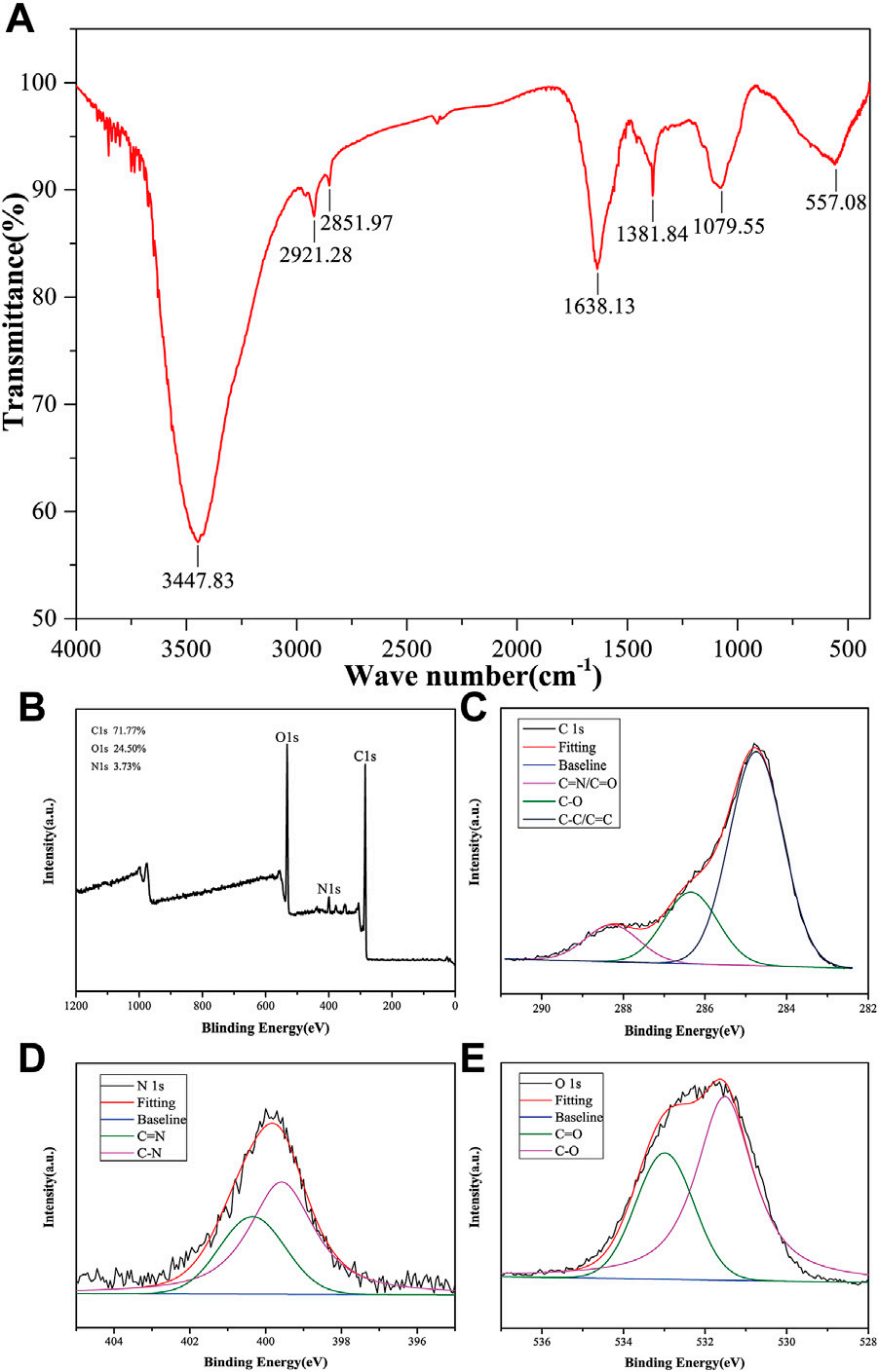
Elemental composition and functional group of CMRC-CDs. **(A)** Fourier transform infrared (FTIR) spectrum of CMRC-CDs. **(B)** Full survey spectrum of X-ray photoelectron spectroscopy (XPS). **(C–E)** High-resolution survey spectra of different elements by XPS.

To gain further insights into the surface functional groups of CMRC-CDs, FTIR spectroscopy was employed, and the corresponding results are presented in Figure 2A. Upon purification, the CMRC-CDs exhibited characteristic peaks at 3,447, 2,921, 2,851, 1,638, 1,381, 1,079, and 557 cm^−1^. The peak observed at 3,447 cm^−1^ can be attributed to the stretching vibrations of O-H and N-H functional groups, whereas the C-H stretching vibrations are discernible through the peaks at 2,921 and 2,851 cm^−1^, respectively (Atchudan et al., 2020). The peak arising at 1,639 cm^−1^ is associated with C=O groups, while the C-H and N-H functional groups give rise to the peak at 1,381 cm^−1^ (Muhammad et al., 2019). Lastly, the peak observed at 1,058 cm^−1^ can be ascribed to the C-O-C absorption vibrations (Muhammad et al., 2019; Wei et al., 2019).

XPS was utilized to execute an elemental analysis of CMRC-CDs; the obtained results revealed that CMRC-CDs primarily consist of carbon (71.77%), oxygen (24.50%), and nitrogen (3.73%). The binding energies corresponding to C 1s, O 1s, and N 1s are delineated by three distinct peaks in Figure 2B, observed respectively at 284.8, 531.8, and 400.0 eV. Three prominent peaks can be seen in the high-resolution C 1s XPS spectra with binding energies of 284.8, 286.4, and 288.3 eV, which are associated with the C-C/C=C, C-O, and C=N/C=O bonds, respectively (Figure 2C) (Godavarthi et al., 2017). Characteristic peaks are visible in the high-resolution O 1s spectra at 531.5 and 533.0 eV, which can be individually attributed to the C-O and C=O functional groups (Figure 2D). In the N 1s spectra, two peaks emerge, corresponding to the N-H and C=N bonds at approximately 399.6 and 400.3 eV, respectively (Figure 2E) (Li et al., 2019).

In conclusion, we conducted a comprehensive investigation of the physical appearance and elemental composition of CMRC-CDs using a combination of techniques, including TEM, HRTEM, TEM mapping, FTIR, and XPS. The consistent results from these methods indicate that the purified CMRC-CDs predominantly consist of carbon, oxygen, and nitrogen elements. The CMRC-CDs feature surface adornment with multiple functional groups, encompassing carbonyl, amino, and hydroxyl entities.

### 2.2 Effect of CMRC-CDs on mCPP-treated mice in the of test

**FIGURE 3:**
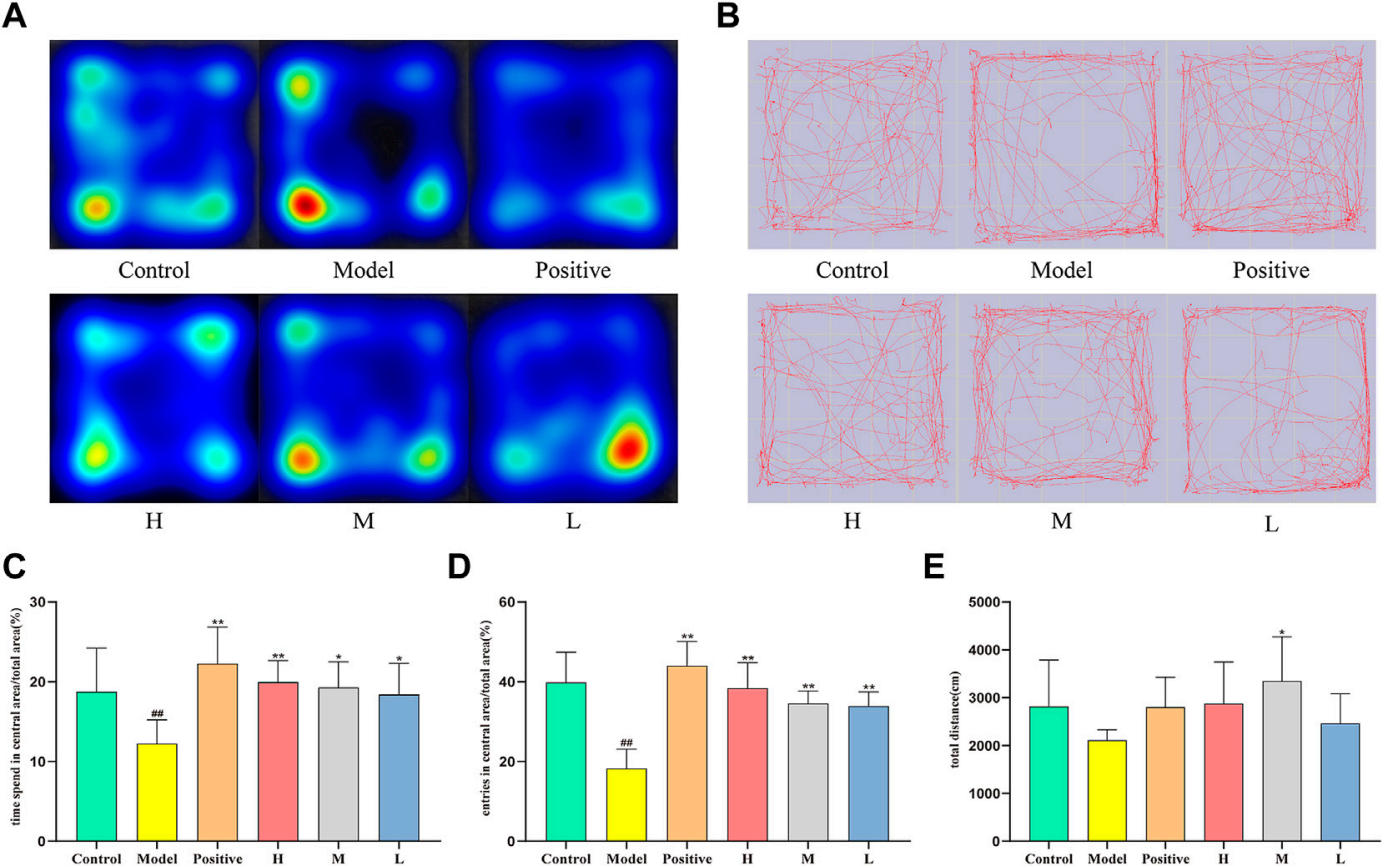
Effect of CMRC-CDs on mCPP-treated mice in the OF test. **(A)** Mean heatmap of mice activity in the OF test. **(B)** Path diagram of mice activity in OF test. **(C)** The proportion of time spent in the central zone relative to the total area (%). **(D)** The percentage of entries made into the central area relative to the total area (%). **(E)** Total distance (cm). The data were presented as the mean ± standard deviation (SD). #*p* < 0.05 and ^##^ *p* < 0.01 vs. control group, ^*^ *p* < 0.05 and ^**^ *p* < 0.01 compared to the model group.

Figure 3 delineates the spatiotemporal dynamics of mice during the open-field (OF) test. As depicted in Figure 3C, compared to the control group, which spent 18.75 ± 5.46% of the time in the central zone, the model group demonstrated a significant reduction, spending only 12.24 ± 2.99% of the time in the same area (*p* < 0.01). In contrast, central zone occupancy increased in the medium- (19.27 ± 3.23%), and low-dose groups (18.39 ± 3.91%) relative to the model group (*p* < 0.05), with a more pronounced enhancement observed in the positive and high-dose groups (*p* < 0.01). Figure 3D presents the frequency of central zone entries as a proportion of total entries across all areas. The model group demonstrated a marked reduction in central zone entry proportion (18.24 ± 4.85%) relative to the control group (39.83 ± 7.62%, *p* < 0.01). Conversely, the positive (43.99 ± 6.09%), high- (38.37 ± 6.39%), medium- (34.55 ± 3.12%), and low-dose groups (33.87 ± 3.59%) all displayed a substantial increment in comparison to the model group (*p* < 0.01). These findings indicate that each dosage administration group effectively increased the inclination of mice to explore and remain in the open central area, as opposed to the model group. This trend is further visually exemplified in the group mean heatmap (Figure 3A) and the movement trajectory map (Figure 3B). Figure 3E reveals the aggregate locomotor distance traversed by the mice, with no significant discrepancies observed among the groups. Consequently, it can be inferred that diazepam and CMRC-CDs modulate the exploratory predilection of mice in the open field without affecting the total distance covered.

### 2.3 Effect of CMRC-CDs on mCPP-treated mice in the EPM test

**FIGURE 4:**
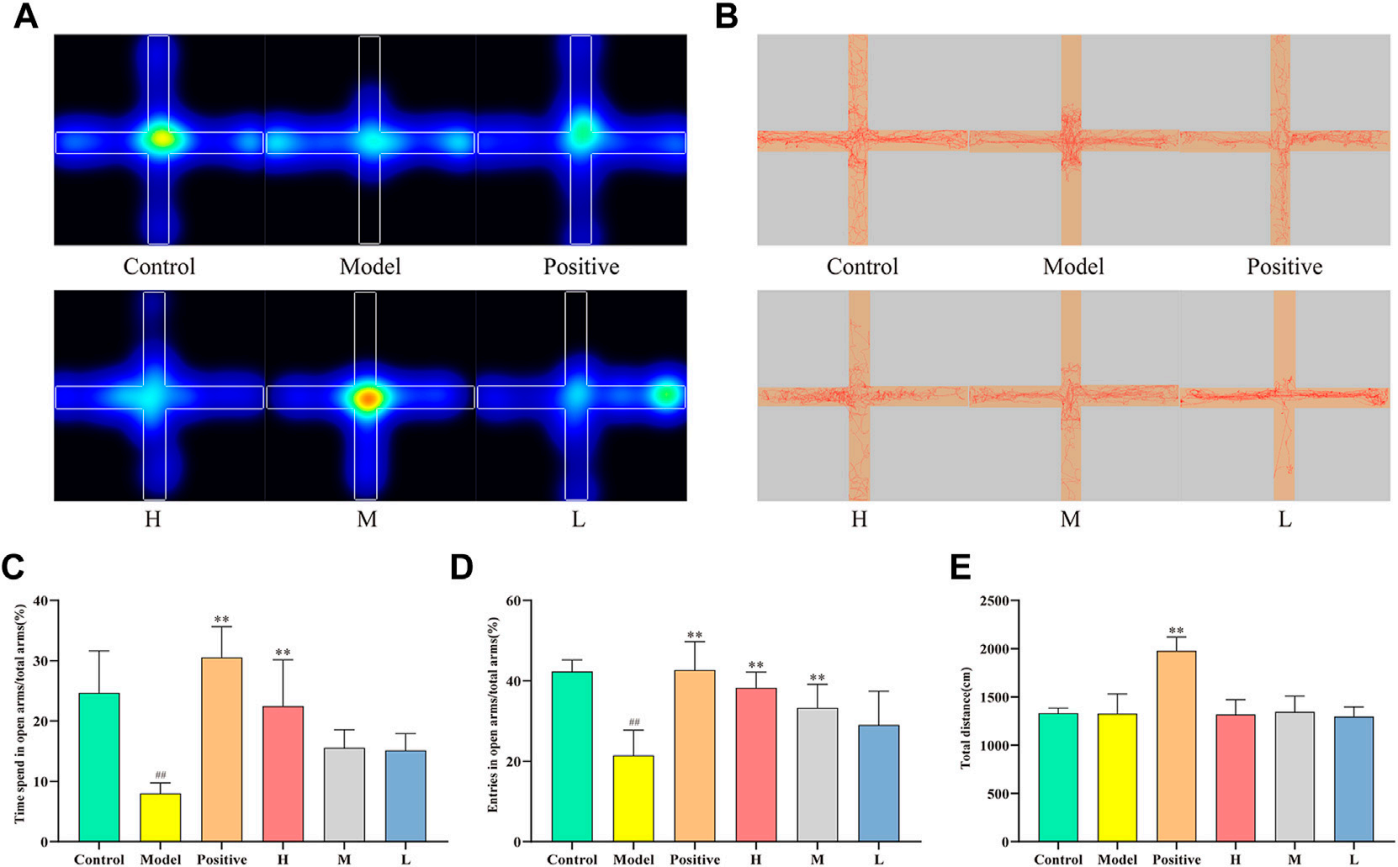
Effect of CMRC-CDs on mCPP-treated mice in the EPM test. **(A)** Mean heatmap of mice activity. **(B)** Path diagram of mice activity in EPM test. **(C)** The proportion of time spent in open arms relative to total arms (%). **(D)** The percentage of entries made into open arms relative to total arms (%). **(E)** Total distance (cm). The data were presented as the mean ± standard deviation. #*p* < 0.05 and ##*p* < 0.01 compared to control group, *p < 0.05 and **p < 0.01 compared to the model group.

Figures 4A, B depict the movement of distinct mouse groups in the elevated cross-maze experiment using mean heatmaps and roadmaps, respectively. The closed arms are represented horizontally, while the open arms are shown vertically. In Figure 4A, color-coded regions delineate mouse movement, with red, yellow, green, and blue indicating the amount of time spent in descending order. The red line in Figure 4B traces the specific path taken by the mice. As observed, the model group’s activity area was considerably smaller compared to the control group, primarily restricted to the closed arms. Conversely, each dosing group broadened their scope of motion and ventured more extensively into open arms. Notably, the positive and high-dose groups demonstrated greater efficacy than the medium- and low-dose groups. Figure 4C reveal that the time spent in open arms was significantly lower in the model group (8.00 ± 1.73%) than in the control group (24.67 ± 6.96%, *p* < 0.01). The positive (30.54 ± 5.09%) and high-dose groups (22.47 ± 7.70%) exhibited significantly higher values than the model group (*p* < 0.01). Although no statistically significant increase was observed in the medium-dose (15.56 ± 3.00%) and low-dose groups (15.13 ± 2.79%) relative to the model group, an upward trend was evident. Figure 4D presents the ratio of open arm entries to total arm entries for each group. The model group (21.47 ± 6.26%) had significantly lower values than the control group (42.31 ± 2.88%, *p* < 0.01). Conversely, the positive (42.66 ± 7.06%), high- (38.25 ± 3.92%), and medium-dose groups (33.29 ± 5.85%) displayed significantly higher values compared to the model group (*p* < 0.01). Despite the lack of statistically significant differences observed between the low-dose group (29.01 ± 8.40%) and the model group, an increasing trend was still apparent. Lastly, Figure 4E illustrates the total distance traveled by each group, with a significant increase observed in the positive group relative to the model group, but no significant differences detected among the remaining groups.

### 2.4 Effects of CMRC-CDs on HPA axis hormones and neurotransmitters in mCPP-treated mice

**FIGURE 5:**
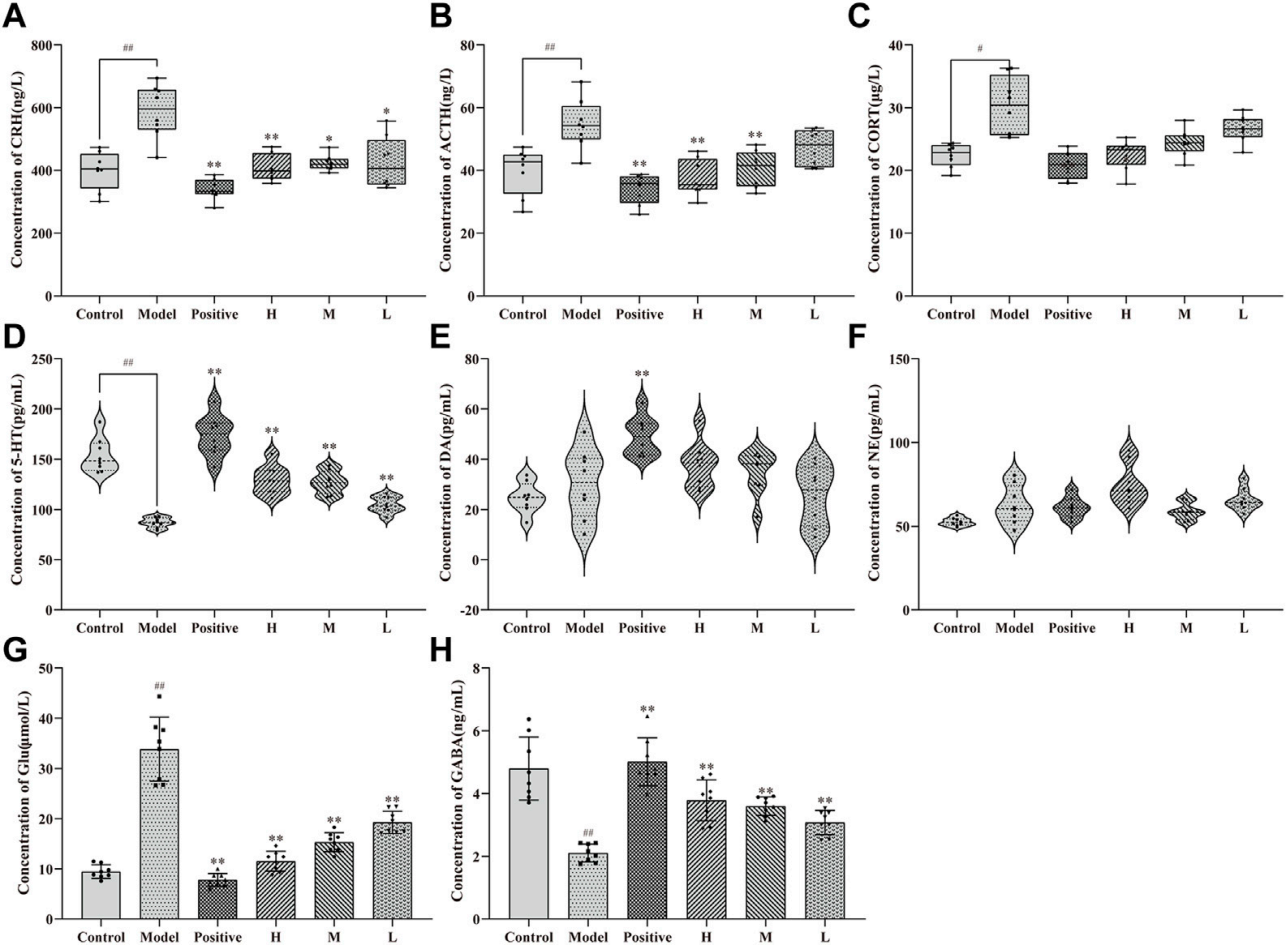
Effect of CMRC-CDs on HPA hormone and Neurotransmitter. The serum concentration of **(A)** CRH, **(B)** ACTH, and **(C)** CORT. Brain tissue homogenization concentration of **(D)** 5-HT **(E)** DA, **(F)** NE, **(G)** Glu, and **(H)** GABA. n = 8/group. ^#^ *p* < 0.05 and ^##^ *p* < 0.01 compared to the control group, ^*^ *p* < 0.05 and ^**^ *p* < 0.01 compared to the model group.

After 14 days of treatment with CMRC-CDs followed by 2 days of behavioral testing, alterations in the levels of HPA axis hormones and neurotransmitters in mice were observed, as illustrated in Figure 5. Specifically, changes in HPA axis hormones in mice serum are demonstrated in Figures 5A–C. In comparison to the control group (CRH: 399.32 ± 60.12 ng/L; ACTH: 39.87 ± 7.42 ng/L; CORT: 22.41 ± 1.82 μg/L), the serum concentrations of CRH, ACTH, and CORT were significantly elevated in the mCPP-treated mice (CRH: 588.71 ± 85.07 ng/L, *p* < 0.01; ACTH: 54.77 ± 7.84 ng/L, *p* < 0.01; CORT: 30.30 ± 4.57 μg/L, *p* < 0.05). Nevertheless, each dosing group demonstrated a varied extent of reduction relative to the model group. Notably, the most pronounced decreasing trend was observed in the positive group (CRH: 339.33 ± 32.93 ng/L, *p* < 0.01; ACTH: 173.88 ± 20.17 ng/L, *p* < 0.01; CORT: 20.89 ± 2.15 μg/L, *p* < 0.01). The high-dose group exhibited a smaller reduction (CRH: 410.75 ± 43.02 ng/L, *p* < 0.01; ACTH: 129.56 ± 14.31 ng/L, *p* < 0.01; CORT: 22.50 ± 2.35 μg/L, *p* < 0.05) in comparison to the positive group. Meanwhile, the group administered a medium dosage (CRH: 423.32 ± 24.61 ng/L, *p* < 0.05; ACTH: 127.15 ± 11.23 ng/L, *p* < 0.01; CORT: 24.44 ± 2.10 μg/L, *p* > 0.05) demonstrated a less pronounced reduction compared to the group receiving a high dosage, and the group administered a low dosage (CRH: 423.89 ± 80.81 ng/L, *p* < 0.05; ACTH: 104.68 ± 8.08 ng/L, *p* < 0.01; CORT: 26.61 ± 2.09 μg/L, *p* > 0.05) exhibited a smaller reduction than the medium-dose group. These findings suggest that CMRC-CDs can decrease serum concentrations of CRH, ACTH, and CORT in mice in a dose-dependent manner.

Figure 5D illustrates the disparities in serum 5-HT concentrations among the groups, revealing a notable reduction in the model group (87.04 ± 5.00 pg/mL, *p* < 0.01) in comparison to the control group (153.68 ± 17.28 pg/mL). Furthermore, a marked elevation was observed in the positive (173.88 ± 20.17 pg/mL, *p* < 0.01), high- (129.56 ± 14.31 pg/mL, *p* < 0.01), medium- (127.15 ± 11.23 pg/mL, *p* < 0.01), and low-dose groups (104.68 ± 8.08 pg/mL, *p* < 0.01) when contrasted with the model group. Figure 5E depicts DA concentrations in the serum of each experimental group, exhibiting no significant divergence between the model and control groups or between the distinct CMRC-CDs concentrations and the model group. Nevertheless, a considerable increase was detected in the positive group relative to the model group (*p* < 0.01). Figure 5F demonstrates that the NE concentrations in the serum of mice from each group did not exhibit significant variations, indicating that the intervention strategy employed in this study did not exert a substantial impact on the NE concentrations of the mice.

**FIGURE 6:**
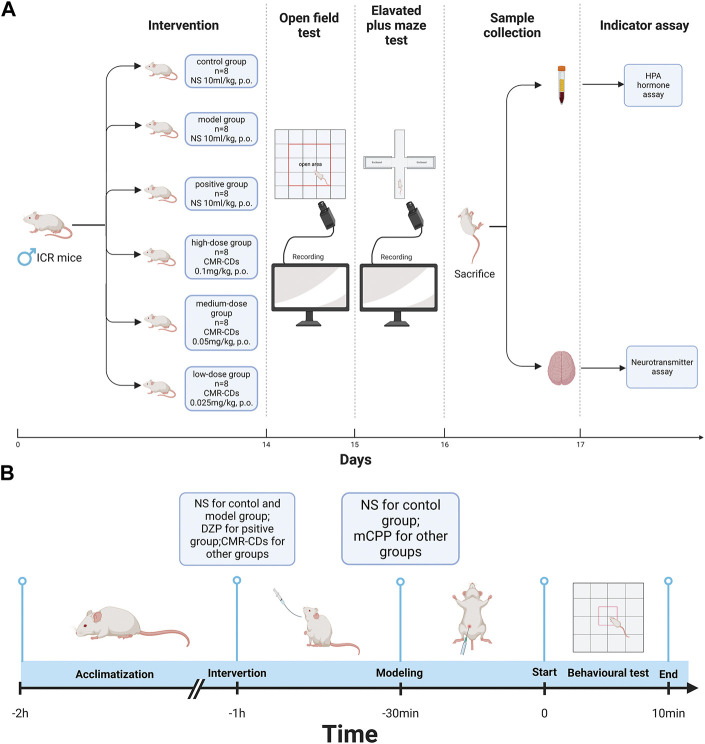
Diagram of the experimental procedure. **(A)** Intervention methods, behavioral testing methods, sample collection, and indicator testing. **(B)** Behavioral testing day process, including site adaptation, gavage intervention, modelling, and behavioral test (Created with BioRender.com).

Figures 5G, H depict the alterations in the concentrations of amino acid neurotransmitters in the brain tissue of mice subjected to various intervention strategies. As illustrated in Figure 5G, the model group exhibited a marked elevation in Glu concentrations (33.85 ± 6.36 μmol/L, *p* < 0.01) compared to the control group (9.45 ± 1.35 μmol/L). Conversely, the positive (7.79 ± 1.27 μmol/L, *p* < 0.01), high- (11.52 ± 1.98 μmol/L, *p* < 0.01), medium- (15.32 ± 1.89 μmol/L, *p* < 0.01), and low-dose groups (19.27 ± 2.23 μmol/L, *p* < 0.01) all demonstrated significantly diminished Glu concentrations relative to the model group. Figure 5H presents an inverse trend for GABA concentrations. Relative to the control group (4.80 ± 1.00 ng/L), the model group displayed a significant reduction in GABA concentrations (2.11 ± 0.29 ng/L, *p* < 0.01). In contrast, the positive (5.01 ± 0.76 ng/L, *p* < 0.01), high- (3.78 ± 0.65 ng/L, *p* < 0.01), medium- (3.60 ± 0.29 ng/L, *p* < 0.01), and low-dose groups (3.08 ± 0.39 ng/L, *p* < 0.01) all exhibited significantly elevated GABA concentrations when compared to the model group. These findings suggest that CMRC-CDs can modulate Glu and GABA concentrations in a dose-dependent manner, resulting in decreased Glu levels and increased GABA levels in the brain tissue of mice. Figure 6 briefly illustrates the entire experimental procedure.

### 2.5 Cytotoxicity of CMRC-CDs

**FIGURE 7:**
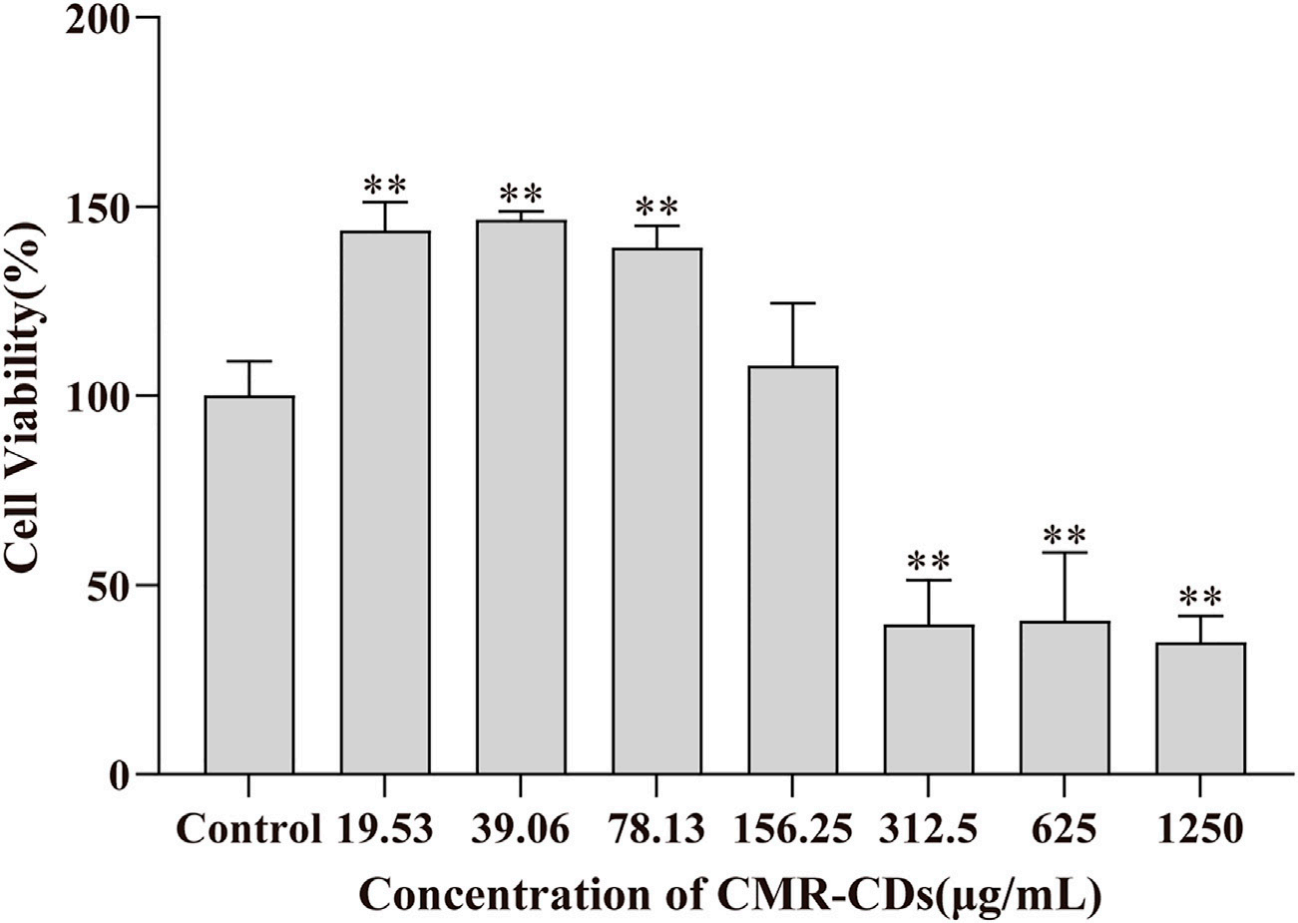
Cell viability for 24 h by CCK-8 method. ^*^ *p* < 0.05 and ^**^ *p* < 0.01 compared to the control group.

In order to assess the cytotoxicity of CMRC-CDs, we employed the murine mononuclear macrophage cell line, RAW 264.7. The cell survival rate was measured after 24 h of exposure to various concentrations of CMRC-CDs, ranging from 19.53 to 1,250 μg/mL, as depicted in Figure 7. Our findings revealed a marked decrease in cell viability at concentrations between 312.5 and 1,250 μg/mL in comparison to the control group (*p* < 0.01), suggesting that CMRC-CDs exert an inhibitory effect on cell proliferation within this concentration range. Interestingly, at a concentration of 156.25 μg/mL, no significant difference in cell viability was observed in relation to the control. Furthermore, cell viability demonstrated a significant enhancement at concentrations between 19.53 and 78.13 μg/mL when compared to the control (*p* < 0.01). Taken together, these results suggest that CMRC-CDs exhibit low cytotoxicity at concentrations below 156.25 μg/mL, indicating their potential biocompatibility for various applications.

## 3 Discussion

Anxiety disorders, representing a predominant health issue in the 21st century, are governed by intricate mechanisms that are not yet fully comprehended. Current understanding implicates neurotransmitters and HPA axis-related hormones in the development of anxiety. Upon HPA axis activation, the hypothalamic paraventricular nucleus synthesizes CRH, which enters the hypophyseal portal blood, subsequently reaching the anterior pituitary to stimulate ACTH synthesis. ACTH, released into the bloodstream, reaches the adrenal cortex and promotes glucocorticoid biosynthesis and release, including corticosterone (Tafet and Nemeroff, 2020). A notable proportion of individuals identified as having chronic anxiety disorders demonstrate heightened activity in the HPA axis (G. E. Tafet et al., 2001; Risbrough and Stein, 2006), suggesting a strong association between elevated HPA axis-related hormones and anxiety (Arborelius et al., 1999; Kinlein et al., 2019), particularly in children experiencing stress-induced events (Faravelli, 2012; Liu and Wang, 2020). Several studies have demonstrated that a variety of anxiolytic drugs, such as tricyclic antidepressants (TCAs), selective serotonin reuptake inhibitors (SSRIs), and benzodiazepines (BZDs), possess the capacity to modulate the hypothalamic-pituitary-adrenal (HPA) axis (Barden et al., 1995; L S Brady et al., 1991; Brady et al., 1992), such as escitalopram’s inhibitory effect on CRH and cortisol (Lenze et al., 2011; Flandreau et al., 2013; Benatti et al., 2018).

Neurotransmitters, another group of substances intimately linked to anxiety production, primarily encompass Glu, GABA, 5-HT, DA, and NE. Glu and GABA, quintessential neurotransmitters within the Central Nervous System (CNS), respectively govern excitatory and inhibitory neurotransmission. Disruptions in excitatory/inhibitory (E/I) balance underpin numerous neuropsychiatric disorders, including anxiety disorders (Prager et al., 2016; Yu et al., 2020). As one of the most phylogenetically ancient neurotransmitters, 5-HT is abundant in the cerebral cortex and synapses, regulating a wide array of brain activities, such as mood modulation (Wirth et al., 2017). Alterations in serotonin 5-HT levels have been shown to substantially impact anxiety-related behaviors (Riedel et al., 2002; Pobbe et al., 2011; Zangrossi and Graeff, 2014), while SSRIs have emerged as first-line therapy for anxiety (H. J. Lee and Stein, 2023). SNRIs, also employed as first-line clinical agents, imply an effect of NE on mood. Hyperactivity of the central noradrenaline system can potentially result in a range of symptoms, such as insomnia, emotional instability, irritability, and anxiety (Yamamoto et al., 2014). According to an expanding corpus of evidence from human brain imaging and preclinical animal research, the mesocorticolimbic dopaminergic system is also suggested to be involved in anxiety disorders (Wee et al., 2008; Zweifel et al., 2011; Russo and Nestler, 2013; Berry et al., 2019).

Due to the complexity and incomplete understanding of anxiety disorder pathogenesis, treating these disorders presents numerous challenges. Current first-line clinical drugs, such as TCAs, SSRIs, and BZDs, are associated with unstable efficacy, significant side effects, or addiction. Consequently, the pursuit of novel anti-anxiety medications remains ongoing. Nanomaterials, characterized by distinct physicochemical properties, have demonstrated promising biological implications in the field of psychiatric disorders (Xue et al., 2016; Ran and Xue, 2018). Furthermore, nanoparticles can traverse the blood-brain barrier (BBB) via receptor-mediated endocytosis, and functionalization or modification facilitates a variety of nanoparticles to cross the BBB through protein and protein-associated receptor interactions (Ran and Xue, 2018). As such, nanoparticles can also serve as drug carriers (Wang et al., 2017). This suggests that nanomedicines may represent potential candidates for the next-generation of anti-anxiety therapeutics.

Traditional herbal medicine, usually derived from natural plants and characterized by its accessibility and lower potential for addiction, represents another promising research avenue. Various herbal medicines have been demonstrated the capacity to ameliorate mood disorders through a range of mechanisms. For instance, Hypericum perforatum exhibits antidepressant properties akin to those of TCAs and SSRIs(Zirak et al., 2019), while Radix rehmanniae extract may exert anxiolytic effects by modulating brain neurotransmitters and neurotrophic proteins (Zhou et al., 2019). Moreover, a specific decoction has been shown to significantly improve cognitive and mood disorders by regulating the GABA/Glu pathway (Xu et al., 2022).

Utilizing various characterization techniques, such as high-resolution electron microscopy, FTIR spectroscopy, and XPS spectroscopy, this study uncovers the presence of carbon dots in Chrysanthemum morifolium Ramat Carbonisata - an herb that has been traditionally used for over 400 years to treat mental illness. The 1.4–4.0 nm diameter of the CMRC-CDs and the abundance of functional groups on their surface suggest their potential as biologically active nanomaterials. Moreover, considering the novelty of these nanoparticles, addressing safety concerns is of paramount importance. CCK-8 assay results demonstrate that CMRC-CDs exhibit negligible toxicity at concentrations below 156.25 μg/mL.

mCPP, a metabolite of trazodone and nefazodone, functions as an agonist for the 5-HT receptor (Pigott et al., 1993; Barbhaiya et al., 1995; Eriksson, 1999). Empirical evidence demonstrates that mCPP injections can modulate the performance of mice in behavioral experiments, and induce anxiety in humans (N. Zhang et al., 2018; Pigott et al., 1993). The OF test is a widely employed paradigm in animal psychology, where subjects are positioned in the center or near the perimeter of the apparatus. In such settings, rodents innately exhibit a preference for navigating the periphery, rather than the central region of the open field. Anxiolytic effects are signified by prolonged time allocation within the central zone and an increased ratio of central area/total activity (Prut and Belzung, 2003). In the present investigation, the square field was subdivided into smaller 5 × 5 squares, designating the central nine squares as the central region. The trajectory of mice within the open field over a 5-min interval was monitored and assessed via video recording. Subsequent calculations were made for the proportion of time spent in the central area relative to the total area, the ratio of entries into the central area to the total area, and the cumulative distance traversed. Our findings reveal that CMRC-CDs substantially enhanced the frequency of mice entering the central region and the proportion of time allocated within this designated area.

In the EPM test, an apparatus comprising four elevated arms arranged in a cross-shaped configuration is utilized to assess anxiety-related behaviors in rodents. The EPM consists of two opposing enclosed arms with walls and two opposing open arms, devoid of any barriers. In the test, experimental mice are placed in the central area of the maze and allowed to explore for a defined short period of time. Given their innate aversion to open or elevated spaces, mice displaying lower anxiety levels will exhibit a higher frequency of open arms visits (Kraeuter, Guest, and Sarnyai, 2019). Our findings demonstrate that the administration of CMRC-CDs results in a significant increase in the proportion of entries to open arms and the duration of time spent in the open arms. Importantly, this effect is dose-dependent, as the observed increase in open arms’ exploration becomes more pronounced with escalating doses of CMRC-CDs. The consistency of the outcomes from both behavioral tests provides strong evidence that CMRC-CDs may ameliorate mCPP-induced anxiety-like behavior in a dose-dependent manner. Notably, there was no substantial difference in the total distance traversed by the mice in either test, implying that CMRC-CDs selectively influence behavioral tendencies without affecting locomotor capabilities. This observation further underscores the potential of CMRC-CDs as a targeted intervention for anxiety-like behaviors in mice.

To elucidate the potential mechanisms underpinning the anxiolytic properties of CMRC-CDs, we quantified the concentrations of HPA axis hormones in serum and neurotransmitters in murine brain tissue. Our data demonstrated a dose-dependent decline in the serum concentrations of all three HPA axis hormones in mice pre-treated with CMRC-CDs. Moreover, compared with the model group, CRH concentrations exhibited significant differences in all three dosage groups, while ACTH levels were significantly different between the high- and medium-dose groups, and CORT levels were significantly different exclusively in the high-dose group. This suggests that the modulatory capacity of CMRC-CDs on HPA axis activity may diminish as their synthesis progresses. Our findings indicate that CMRC-CDs may exert anxiolytic effects by reducing HPA axis hormone concentrations, with the principal site of regulation potentially being the CRH synthesis in the hypothalamus.

Investigations into neurotransmitter concentrations revealed a decline in Glu levels alongside a concurrent elevation in GABA concentrations within cerebral tissues of the mice administered with CMRC-CDs. A noteworthy influence was observed across all three dosages. These findings suggest that CMRC-CDs may contribute to the alleviation of mood disorders by modulating the Glu/GABA pathway. Considering that GABA can be synthesized via the decarboxylation of Glu (Sarasa et al., 2020), a plausible hypothesis is that CMRC-CDs may facilitate this decarboxylation process, thus promoting the reestablishment of E/I homeostasis.

In a comparative analysis of the three primary monoamine neurotransmitters, CMRC-CDs exhibited a significant increase in 5-HT concentrations in relation to the model group. This elevation was statistically significant across all three administered dosages. In contrast, the administration of various CMRC-CDs doses did not produce significant effects on DA and NE concentrations. Considering that DA is posited to maintain a close relationship with exercise capacity (Meeusen and De Meirleir, 1995; Dohnalová et al., 2022), these findings are consistent with the results of the behavioral experiments, wherein no significant differences were observed in the total distance traversed.

## 4 Conclusion

In summary, we have successfully synthesized carbon dots derived from Chrysanthemum morifolium Ramat (CMRC-CDs) that exhibit exceptional fluorescence properties and a surface abundant in functional groups. Our thorough behavioral investigations demonstrated that CMRC-CDs effectively ameliorate mCPP-induced anxiety-like behavior in murine models in a dose-responsive manner. This therapeutic effect is primarily mediated through the modulation of HPA axis hormone levels, amino acid neurotransmitter concentrations, and serotonin 5-HT levels. Owing to their environmentally benign nature, cost-effectiveness, and facile preparation, CMRC-CDs hold immense potential as novel nanomedicines for the management of anxiety disorders. Furthermore, our findings offer valuable insights into the therapeutic prospects of traditional Chinese herbal medicine for treating mood disorders, which could inspire further investigations in this field.

## 5 Materials and methods

### 5.1 Chemicals

CMR was purchased from Beijing Qiancao Herbal Pieces Co., Ltd. (Beijing, China). Diazepam (DZP) tablets were obtained from Beijing Yimin Pharmaceutical Factory (Beijing, China). Dialysis membranes with 1,000 Da molecular weight cutoff (MWCO) were provided by Beijing Ruida Henghui Technology Development Co., Ltd. (Beijing, China). ELISA kits for measuring neurotransmitter and HPA hormone concentrations were purchased from Jiangsu Kete Biotechnology Co., Ltd. (Jiangsu, China). The cell counting kit-8 (CCK-8) was acquired from Dojindo Molecular Technologies, Inc. (Kumamoto, Japan). Dulbecco’s Modified Eagle’s Medium (DMEM), fetal bovine serum (FBS), and antibiotics were sourced from Gibco BRL (Gaithersburg, MD, United States). Deionized water (DW) was used in all experiments.

### 5.2 Animals and cells

All mice were maintained under standardized conditions, including *ad libitum* access to food and water, an ambient temperature of 25.0°C ± 1.0°C, a relative humidity ranging from 55%–65%, and a diurnal rhythm of 12 h light and 12 h darkness. Behavioral experiments were carried out in a tranquil laboratory environment between the hours of 09:00 and 15:00. RAW 264.7 cells were employed in cell viability assays due to their ease of culture and expansion, as well as their ability to maintain high proliferation capacity and *in vitro* cell viability. Furthermore, RAW 264.7 cells exhibit pronounced sensitivity to stimulating substances and promptly respond to external stimuli, eliciting diverse biological effects.

### 5.3 Preparation of CMRC-CDs

**FIGURE 8:**
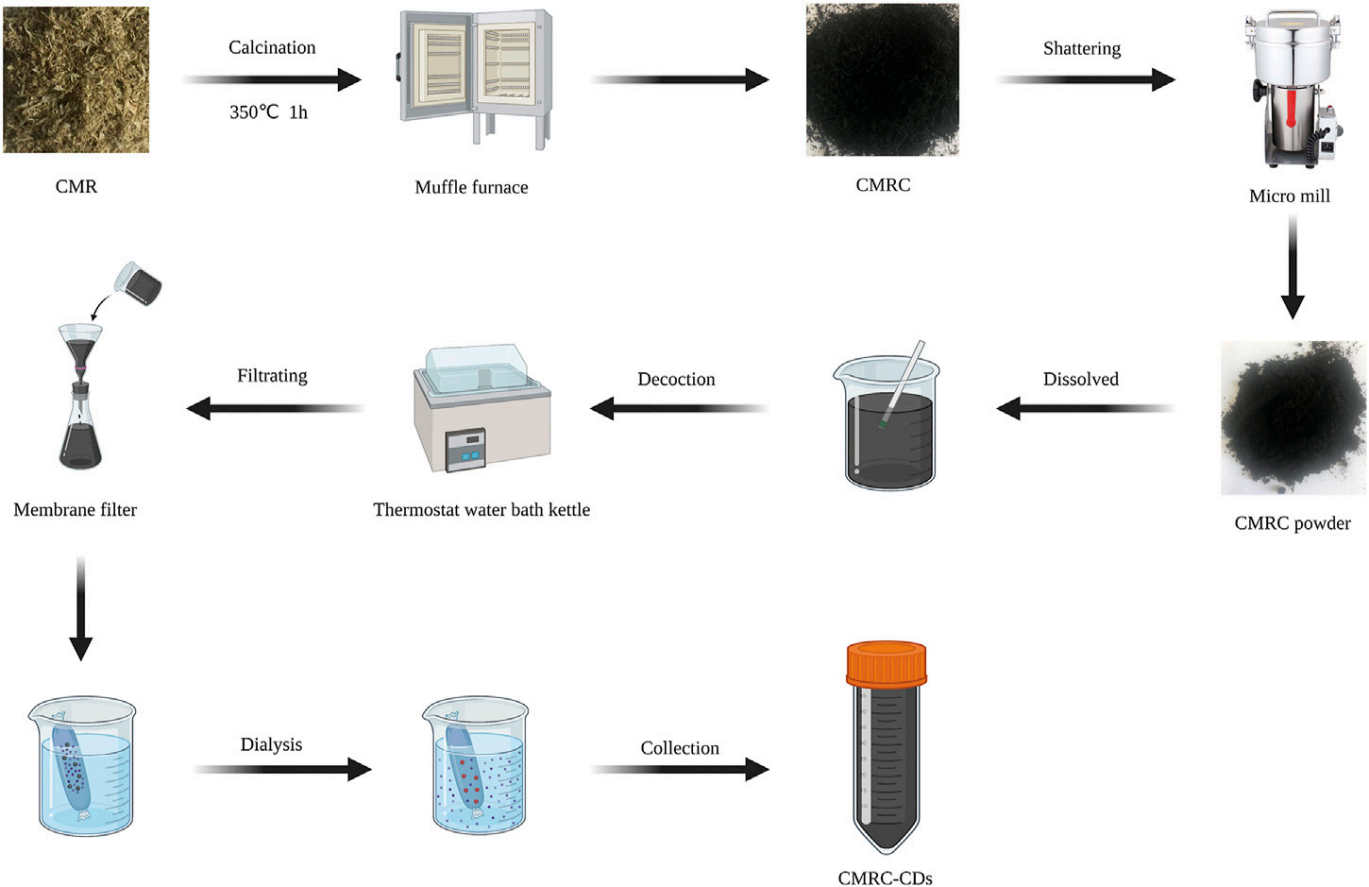
A procedural schematic represents the synthesis of carbon dots obtained from Chrysanthemum morifolium Ramat (CMRC-CDs) (Created with BioRender.com).

The synthesis of CMRC-CDs was achieved utilizing CMR as the carbon source. In the initial stage, CMR was situated in hermetically sealed porcelain crucibles and subjected to carbonization at a temperature of 350°C for a duration of 1 h, utilizing a muffle furnace. After cooling down to ambient temperature, the resulting CMRC was pulverized into fine fragments using a micro mill. The fine CMRC powder was subsequently dispersed in deionized water at a 1:30 ratio and heated to 100°C for three 1-h boiling sessions. The mixture was then filtered through a 0.22 µm microfiltration membrane. Following this, the solution was dialyzed against deionized water for 7 days using a dialysis membrane with a 1,000 Da molecular weight cut-off, changing the dialysis solution every 8 h. Afterward, the CMRC-CDs solution was placed in a refrigerator at 4°C so that it could be utilized in the future. Figure 8 illustrates a schematic diagram outlining the preparation process.

### 5.4 Characterization of CMRC-CDs

The physical structure and morphology of CMRC-CDs were meticulously investigated and quantified using transmission electron microscopy (TEM) and high-resolution TEM (HRTEM) at an accelerating voltage of 200 kV. The X-ray Diffractometer was employed to acquire X-ray diffraction (XRD) patterns. The photoluminescence characteristics of CMRC-CDs were scrutinized utilizing a fluorescence spectrophotometer, while the ultraviolet-visible (UV-vis) absorption spectra were probed with a UV-vis spectrometer. In the range of 400–4,000 cm^−1^, Fourier transform infrared (FTIR) spectroscopy was employed to analyze the organic functional groups present in the CMRC-CDs. Additionally, X-ray photoelectron spectroscopy (XPS) was utilized to determine elemental characterization of the CMRC-CDs.

### 5.5 Open-field (OF) test

The OF test was employed to assess the autonomous behavior, exploratory tendencies, and anxiety levels of experimental animals when introduced to a novel environment. The open-field apparatus consists of a reaction chamber measuring 30 cm in height, featuring a square base with dimensions of 50 cm × 50 cm, a black interior, and a floor partitioned evenly into 25 smaller squares. The experiments are meticulously documented using a high-resolution camera positioned directly above the experimental apparatus. This camera is operated by a sophisticated computer program, ensuring accuracy and consistency in data capture. The peripheral area comprised 16 zones situated adjacent to the walls; the central area consisted of the remaining nine central zones. In the experimental setup, every mouse was carefully positioned at the center of the container and subsequently granted a 10-min period for exploration. Prior to testing, animals were acclimated to the experimental environment for 60 min. To mitigate the potential confounding influence of olfactory cues from prior experimental subjects, the apparatus underwent a rigorous cleaning procedure with a 75% ethanol solution following each trial, aimed at eliminating residual odors. This measure was taken to minimize the risk of contamination and reduce the possibility of spurious experimental outcomes stemming from olfactory stimuli.

Anxiety levels and exploratory behavior were assessed by quantifying the proportion of time spent in the central area, the proportion of entries in the central area, and the total distance traveled, respectively (Carola et al., 2002; Saitoh et al., 2004).

### 5.6 Elevated plus maze (EPM) test

The primary components of the Elevated plus maze (EPM) apparatus consist of two opposing closed arms (30 cm × 5 cm) and two opposing non-transparent open arms (30 cm × 5 cm × 25 cm), arranged in a cross-shaped configuration. The apparatus is elevated to a height of 50 cm from the ground, with the arms connected to a central platform measuring 5 cm × 5 cm. The experiments are meticulously documented using a high-resolution camera positioned directly above the experimental apparatus. This camera is operated by a sophisticated computer program, ensuring accuracy and consistency in data capture. Every murine subject is delicately situated at the core of the apparatus, oriented towards an open arm, and meticulously monitored for an interval of 10 min. Prior to testing, animals are acclimated to the experimental environment for a period of 2 h. To mitigate potential bias from olfactory cues left by previous subjects, the apparatus is thoroughly cleaned with a 75% ethanol solution after each trial (Guo et al., 2011).

Parameters indicative of anxiolytic-like behavior, such as the percentage of time spent in open arms [(open arm duration/total duration) × 100] and the proportion of open arm entries [(open arms entries/total entries) × 100], are assessed through analysis of the recorded video footage. To gauge alterations in exploratory activity, the total distance traversed by the subjects is calculated. Any mouse that inadvertently falls out of the maze is excluded from the experiment.

### 5.7 Sample collection

Mice were granted a 24-h recovery period following the behavioral assessments before blood collection commenced. Blood samples were procured employing serum collection tubes, subsequently permitting coagulation for a duration of 2 h at room temperature. Following this, samples were subjected to centrifugation at 3,000 revolutions per minute and 4°C for a 10-min interval, resulting in the isolation of the serum component. Subsequent to the blood sampling, the mice, under anesthesia, were euthanized via decapitation. Their brains were then delicately excised on ice, with rigorous care taken to mitigate any potential tissue damage. For subsequent analysis of the relevant variables, samples were preserved at −80°C.

### 5.8 Quantification of neurotransmitter

Neurotransmitter concentrations, encompassing 5-HT, NE, DA, GABA, and Glu, were ascertained from brain tissue samples through the implementation of ELISA. Murine ELISA kits were employed in strict adherence to the guidelines provided by the manufacturer. The samples were measured for optical density (OD) with a wavelength of 450 nm, utilizing a microplate spectrophotometer (Biotek, VT, United States). Subsequently, neurotransmitter concentrations were denoted in units of either ng/mL or pg/mL, as appropriate.

### 5.9 Quantification of HPA hormone

ELISA was employed for the quantification of HPA axis hormones, including CRH, ACTH, and CORT, in serum samples. Adherence to the manufacturer’s guidelines for the utilization of murine ELISA kits was ensured. Optical density (OD) measurements were performed at 450 nm.

### 5.10 Cell viability assay of CMRC-CDs

In this investigation, RAW 264.7 cells were propagated in DMEM with 10% FBS, 100 mg/mL streptomycin, and 100 IU/mL penicillin, followed by an incubation period at 37°C within a humidified environment containing 5% CO_2_. The cytotoxic effects of CMRC-CDs on RAW 264.7 cells were evaluated employing the CCK-8 assay (Jia et al., 2017). Cells were cultured in 96-well plates at a density of 1 × 10^5^ cells/mL employing serum-free media, followed by a 24-h incubation period under standard conditions. Next, the cells were treated with various concentrations of CMRC-CDs (1,250, 625, 312.5, 156.25, 78.13, and 39.06 μg/mL) by adding 100 μL of the solution to the corresponding wells, followed by a 24-h incubation period. Upon the elimination of the culture media and subsequent dual rinsing with PBS, each well was supplemented with 10 μL of CCK-8 solution, followed by incubation of the cells for an additional 4 h. The OD of each well was measured at a 450 nm wavelength utilizing a microplate reader. The relative cell viability was determined by calculating the percentage relative to the control group using the following formula:

$$Cell\;viability\;\left(\%\;of\;control\right)=\frac{A_{e}-A_{b}}{A_{c}-A_{b}}\times 100$$ (1)

The absorbance values for the experimental, blank, and control groups are denoted as Ae, Ab, and Ac, respectively.

### 5.11 Experimental procedure

A total of 48 male ICR mice were randomly and equally divided into six groups. After 3 days of acclimatization, the intervention protocol for each group began as follows: control group, model group, and positive group (normal saline [NS] 10 mL/kg, i.g.), high-dose pretreatment group (CMRC-CDs 0.1 mg/kg, i.g.), medium-dose pretreatment group (CMRC-CDs 0.05 mg/kg, i.g.), and low-dose pretreatment group (CMRC-CDs 0.025 mg/kg, i.g.). The intervention was performed for 14 consecutive days.

The OF test was conducted on day 15 and the EPM test was conducted on day 16, respectively. The mice were brought to the experimental site 2 h before the start of the experiment for acclimatization. One hour before the experiment began, each group received the following interventions: control group (NS 10 mL/kg, i.g.), model group (NS 10 mL/kg, i.g.), positive group (DZP, 2 mg/kg, i.g.), high-dose pretreatment group (CMRC-CDs 0.1 mg/kg, i.g.), medium-dose pretreatment group (CMRC-CDs 0.05 mg/kg, i.g.), and low-dose pretreatment group (CMRC-CDs 0.025 mg/kg, i.g.). Anxiety models were established 30 min before the experiment using the following protocols: control group (NS 10 mL/kg, i.p.), other groups (mCPP 2 mg/kg, i.p.) (N. Zhang et al., 2018).

After the EPM test, mice were fasted for 24 h with *ad libitum* access to water. Samples were collected on day 17. Mice received the intervention 1 h after arriving at the experimental site. Anxiety models were established 30 min later. Following an additional 30-min period, the mice were humanely euthanized via decapitation, after which blood samples and brain tissue were systematically collected. The intervention methods and anxiety model establishment were the same as during the behavioral experiments.

Following sample collection, HPA axis hormone concentrations in serum and neurotransmitter concentrations in brain tissue were determined using ELISA. Video analysis software was used to observe and extract data from videos of the mouse behavioral experiments. For the OF test, observations included time spent in the central area, the number of entries into the central area, the total number of entries into all areas, and the total distance traveled. For the EPM test, the metrics observed included dwell time in open arms, total dwell time in the open and closed arms, number of entries into open arms, the total number of entries into the open and closed arms, and total distance traveled.

### 5.12 Statistical analysis

Statistical analyses were performed using SPSS 20.0. For data adhering to normal distribution with equal variances, means and standard deviations were computed. Multiple comparisons were carried out using one-way analysis of variance (ANOVA). Non-normally distributed data were presented as the median (interquartile range). A non-parametric test was employed for within-group comparisons of such data, whereas between-group differences were examined with the Kruskal–Wallis test. A *p*-value of less than 0.05 was deemed to indicate statistical significance.

## Data Availability

The original contributions presented in the study are included in the article/Supplementary Material, further inquiries can be directed to the corresponding authors.

## References

[B1] Alawdi S. H. El-Denshary E. S. Safar M. M. Eidi H. David M. O. MosaadAbdel-Wahhab A. (2017). Neuroprotective effect of nanodiamond in alzheimer’s disease rat model: A pivotal role for modulating NF-?b and STAT3 signaling. Mol. Neurobiol. 54 (3), 1906–1918. 10.1007/s12035-016-9762-0 26897372

[B2] Arborelius L. Owens M. Plotsky P. Nemeroff C. (1999). The role of corticotropin-releasing factor in depression and anxiety disorders. J. Endocrinol. 160 (1), 1–12. 10.1677/joe.0.1600001 9854171

[B3] Ashrafizadeh M. Mohammadinejad R. Kumar Kailasa S. Ahmadi Z. Elham Ghasemipour A. Pardakhty A. (2020). Carbon dots as versatile nanoarchitectures for the treatment of neurological disorders and their theranostic applications: A review. Adv. Colloid Interface Sci. 278, 102123. 10.1016/j.cis.2020.102123 32087367

[B4] Atchudan R. Thomas Nesakumar J. I. E. Mani S. Perumal S. Vinodh R. Thirunavukkarasu S. (2020). Facile synthesis of a novel nitrogen-doped carbon dot adorned zinc oxide composite for photodegradation of methylene blue. Dalton Trans. 49 (48), 17725–17736. 10.1039/D0DT02756A 33237044

[B5] Bala Areeg Hoang Minh T. N. Wayne J. Hellstrom G. (2018). Post-SSRI sexual dysfunction: A literature review. Sex. Med. Rev. 6 (1), 29–34. 10.1016/j.sxmr.2017.07.002 28778697

[B6] Barbhaiya R. H. Shukla U. A. Natarajan C. S. Behr D. A. Greene D. S. Sainati S. M. (1995). Single- and multiple-dose pharmacokinetics of nefazodone in patients with hepatic cirrhosis. Clin. Pharmacol. Ther. 58 (4), 390–398. 10.1016/0009-9236(95)90051-9 7586930

[B7] Barden N. Reul J. M. H. M. Holsboer F. (1995). Do antidepressants stabilize mood through actions on the hypothalamic-pituitary-adrenocortical system? Trends Neurosci. 18 (1), 6–11. 10.1016/0166-2236(95)93942-Q 7535490

[B8] Benatti C. Alboni S. Blom J. M. C. Mendlewicz J. Tascedda F. Brunello N. (2018). Molecular changes associated with escitalopram response in a stress-based model of depression. Psychoneuroendocrinology 87, 74–82. 10.1016/j.psyneuen.2017.10.011 29049934

[B9] Berry A. S. White R. L. Furman D. J. Naskolnakorn J. R. Shah V. D. Esposito M. D. (2019). Dopaminergic mechanisms underlying normal variation in trait anxiety. J. Neurosci. 39 (14), 2735–2744. 10.1523/JNEUROSCI.2382-18.2019 30737306 PMC6445999

[B10] Brady L. S. Whitfield H. J. Fox R. J. Gold P. W. Herkenham M. (1991). Long-term antidepressant administration alters corticotropin-releasing hormone, tyrosine hydroxylase, and mineralocorticoid receptor gene expression in rat brain. Therapeutic implications. J. Clin. Investigation 87 (3), 831–837. 10.1172/JCI115086 PMC329870 1671867

[B11] Brady L. S. Gold P. W. Herkenham M. Lynn A. B. Whitfield H. J. (1992). The antidepressants fluoxetine, idazoxan and phenelzine alter corticotropin-releasing hormone and tyrosine hydroxylase MRNA levels in rat brain: Therapeutic implications. Brain Res. 572 (1–2), 117–125. 10.1016/0006-8993(92)90459-M 1351783

[B12] Carola V. D’Olimpio F. Brunamonti E. Mangia F. Renzi P. (2002). Evaluation of the elevated plus-maze and open-field tests for the assessment of anxiety-related behaviour in inbred mice. Behav. Brain Res. 134 (1–2), 49–57. 10.1016/S0166-4328(01)00452-1 12191791

[B13] Chen Z. Ye S. Y. Yang Y. Li Z. Y. (2019). A review on charred traditional Chinese herbs: Carbonization to yield a haemostatic effect. Pharm. Biol. 57 (1), 498–506. 10.1080/13880209.2019.1645700 31401925 PMC6713113

[B14] Chu F. Li K. Li X. Xu L. Huang J. Yang Z. (2021). Graphene oxide ameliorates the cognitive impairment through inhibiting PI3K/akt/MTOR pathway to induce autophagy in AD mouse model. Neurochem. Res. 46 (2), 309–325. 10.1007/s11064-020-03167-z 33180247

[B15] Cui L. Ren X. Sun M. Liu H. Xia L. (2021). Carbon dots: Synthesis, properties and applications. Nanomaterials 11 (12), 3419. 10.3390/nano11123419 34947768 PMC8705349

[B16] Delpino F. M. Nascimento da Silva C. Santos Jerônimo J. Stark Mulling E. Leal da Cunha L. Krause Weymar M. (2022). Prevalence of anxiety during the COVID-19 pandemic: A systematic review and meta-analysis of over 2 million people. J. Affect. Disord. 318 272–282. 10.1016/j.jad.2022.09.003 36096370 PMC9462928

[B17] Dohnalová L. Lundgren P. JamieCarty R. E. Goldstein N. Wenski S. L. Nanudorn P. (2022). A microbiome-dependent gut–brain pathway regulates motivation for exercise. Nature 612 (7941), 739–747. 10.1038/s41586-022-05525-z 36517598 PMC11162758

[B18] Durán N. Simões M. B. de Moraes A. C. M. Fávaro W. J. Seabra A. B. (2016). Nanobiotechnology of carbon dots: A review. J. Biomed. Nanotechnol. 12 (7), 1323–1347. 10.1166/jbn.2016.2225 29336531

[B19] Eriksson E. Engberg G. Bing O. Nissbrandt H. (1999). Effects of MCPP on the extracellular concentrations of serotonin and dopamine in rat brain. Neuropsychopharmacology 20 (3), 287–296. 10.1016/S0893-133X(98)00070-0 10063489

[B20] Faravelli C. Lo Sauro C. Godini L. Lelli L. Benni L. Pietrini F. (2012). Childhood stressful events, HPA Axis and anxiety disorders. World J. Psychiatry 2 (1), 13–25. 10.5498/wjp.v2.i1.13 24175164 PMC3782172

[B21] Flandreau E. I. Chase H. Bourke K. J. R. Vale W. W. Nemeroff C. B. Owens M. J. Owens M. J. (2013). Escitalopram alters gene expression and HPA Axis reactivity in rats following chronic overexpression of corticotropin-releasing factor from the central amygdala. Psychoneuroendocrinology 38 (8), 1349–1361. 10.1016/j.psyneuen.2012.11.020 23267723 PMC3749072

[B22] GBD 2019 Mental Disorders Collaborators (2022). Global, regional, and national burden of 12 mental disorders in 204 Countries and Territories, 1990–2019: A systematic analysis for the global burden of disease study 2019. Lancet Psychiatry 9 (2), 137–150. 10.1016/S2215-0366(21)00395-3 35026139 PMC8776563

[B23] Godavarthi S. Mohan Kumar K. Vázquez Vélez E. Hernandez-Eligio A. Mahendhiran M. Hernandez-Como N. (2017). Nitrogen doped carbon dots derived from sargassum fluitans as fluorophore for DNA detection. J. Photochem. Photobiol. B Biol. 172 36–41. 10.1016/j.jphotobiol.2017.05.014 28514712

[B24] Guo J. Y. Yuan X. Y. Sui F. Zhang W. C. Wang J. Y. Luo F. (2011). Placebo analgesia affects the behavioral despair tests and hormonal secretions in mice. Psychopharmacology 217 (1), 83–90. 10.1007/s00213-011-2259-7 21448649

[B25] Henna T. K. Raphey V. R. Sankar Renu Shirin V. K. A. Gangadharappa H. V. Pramod K. (2020). Carbon nanostructures: The drug and the delivery system for brain disorders. Int. J. Pharm. 587 119701. 10.1016/j.ijpharm.2020.119701 32736018

[B26] Horowitz M. A. Taylor D. (2019). Tapering of SSRI treatment to mitigate withdrawal symptoms. Lancet Psychiatry 6 (6), 538–546. 10.1016/S2215-0366(19)30032-X 30850328

[B27] Jacobson Lauren (2014). Hypothalamic-pituitary-adrenocortical Axis: Neuropsychiatric aspects. Compr. Physiol. 4, 715–738. 10.1002/cphy.c130036 24715565

[B28] Jaleel J. A. Pramod K. (2018). Artful and multifaceted applications of carbon dot in biomedicine. J. Control. Release 269 302–321. 10.1016/j.jconrel.2017.11.027 29170139

[B29] Jia P. Yu L. Tao C. Guo D. Zhang Z. Liu S. (2017). Chitosan oligosaccharides protect nucleus pulposus cells from hydrogen peroxide-induced apoptosis in a rat experimental model. Biomed. Pharmacother. 93 807–815. 10.1016/j.biopha.2017.06.101 28715865

[B30] Khayal A. Dawane V. Amin M. A. Tirth V. Yadav V. K. (2021). “Advances in the methods for the synthesis of carbon dots and their emerging applications.” Polymers 13 (18): 3190. 10.3390/polym13183190 34578091 PMC8469539

[B31] Kinlein S. A. Phillips D. J. Keller C. R. Karatsoreos I. N. (2019). Role of corticosterone in altered neurobehavioral responses to acute stress in a model of compromised hypothalamic-pituitary-adrenal Axis function. Psychoneuroendocrinology 102 248–255. 10.1016/j.psyneuen.2018.12.010 30594817 PMC7649055

[B32] Kraeuter A. K. Guest P. C. Sarnyai Z. (2019). “The elevated plus maze test for measuring anxiety-like behavior in rodents,” in Pre-clinical models. Methods in molecular biology. Editor Guest Paul C. (New York, NY: Springer New York), 69–74. 10.1007/978-1-4939-8994-2_4 30535682

[B33] Lee H. J. Stein M. B. (2023). Update on treatments for anxiety-related disorders. Curr. Opin. Psychiatry 36 (2), 140–145. 10.1097/YCO.0000000000000841 36480651

[B34] Lee S. Rhee D. K. (2017). Effects of ginseng on stress-related depression, anxiety, and the hypothalamic–pituitary–adrenal Axis. J. Ginseng Res. 41 (4), 589–594. 10.1016/j.jgr.2017.01.010 29021708 PMC5628357

[B35] Lenze E. J. Mantella R. C. Shi P. Goate A. M. Nowotny P. Butters M. A. (2011). Elevated cortisol in older adults with generalized anxiety disorder is reduced by treatment: A placebo-controlled evaluation of escitalopram. Am. J. Geriatric Psychiatry 19 (5): 482–490. 10.1097/JGP.0b013e3181ec806c PMC3424606 20808146

[B36] Li D. Xu K.Y. Zhao W. P. Liu M. F. Feng R. De-qiang L. (2022). Chinese medicinal herb-derived carbon dots for common diseases: Efficacies and potential mechanisms. Front. Pharmacol. 13 815479. 10.3389/fphar.2022.815479 35281894 PMC8906921

[B37] Li D. Na X. Zhou W. Wang C. Li Y. Zhu B. W. (2019). Adverse effects of fluorescent carbon dots from canned yellow croaker on cellular respiration and glycolysis. Food & Funct. 10 (2), 1123–1131. 10.1039/C8FO02602E 30724933

[B38] Liu L. Wang M. (2020). Parental corporal punishment and child anxiety in China: The moderating role of HPA-Axis activity. J. Affect. Disord. 273 500–507. 10.1016/j.jad.2020.04.055 32560946

[B39] Liu L. Liu C. Wang Y. Wang P. Li Y. Bingjin L. (2015). Herbal medicine for anxiety, depression and insomnia. Curr. Neuropharmacol. 13 (4), 481–493. 10.2174/1570159x1304150831122734 26412068 PMC4790408

[B40] Luo W. K. Zhang L. L. Yang Z. Y. Guo X. H. Wu Y. Zhang W. (2021). Herbal medicine derived carbon dots: Synthesis and applications in therapeutics, bioimaging and sensing. J. Nanobiotechnology 19 (1), 320. 10.1186/s12951-021-01072-3 34645456 PMC8513293

[B41] Mancuso C. E. Tanzi M. G. Gabay M. (2004). Paradoxical reactions to benzodiazepines: Literature review and treatment options. Pharmacotherapy 24 (9), 1177–1185. 10.1592/phco.24.13.1177.38089 15460178

[B42] Mansuriya B. D. Zeynep A. (2021). Carbon dots: Classification, properties, synthesis, characterization, and applications in health care—an updated review (2018–2021). Nanomaterials 11 (10), 2525. 10.3390/nano11102525 34684966 PMC8541690

[B43] Meeusen R. De Meirleir K. (1995). Exercise and brain neurotransmission. Sports Med. 20 (3), 160–188. 10.2165/00007256-199520030-00004 8571000

[B44] Meldrum B. S. (2000). Glutamate as a neurotransmitter in the brain: Review of physiology and pathology. J. Nutr. 130 (4), 1007S–1015S. 10.1093/jn/130.4.1007S 10736372

[B45] Muhammad W. Ullah N. Haroon M. Abbasi B. H. (2019). Optical, morphological and biological analysis of zinc oxide nanoparticles (ZnO NPs) using *papaver somniferum* L. RSC Adv. 9 (51), 29541–29548. 10.1039/C9RA04424H 35531532 PMC9071912

[B46] Olivier J. D. A. Olivier. B. (2020). “Translational studies in the complex role of neurotransmitter systems in anxiety and anxiety disorders,” in Anxiety disorders. Advances in experimental medicine and biology. Editor Yong-Ku K. (Singapore: Springer Singapore), 1191, 121–140. 10.1007/978-981-32-9705-0_8 32002926

[B47] Panossian A. Wikman G. Sarris J. (2010). Rosenroot (Rhodiola rosea): Traditional use, chemical composition, pharmacology and clinical efficacy. Phytomedicine 17 (7), 481–493. 10.1016/j.phymed.2010.02.002 20378318

[B48] Petursson H. (1994). The benzodiazepine withdrawal syndrome. Addiction 89 (11), 1455–1459. 10.1111/j.1360-0443.1994.tb03743.x 7841856

[B49] Pigott T. A. James L. Hill T. A. G. L’Heureux F. Bernstein S. Rubenstein C. S. (1993). A comparison of the behavioral effects of oral versus intravenous MCPP administration in OCD patients and the effect of metergoline prior to IV MCPP. Biol. Psychiatry 33 (1), 3–14. 10.1016/0006-3223(93)90272-F 8420593

[B50] Pobbe R. L. H. Zangrossi H. Caroline Blanchard D. Blanchard R. J. (2011). Involvement of dorsal raphe nucleus and dorsal periaqueductal gray 5-HT receptors in the modulation of mouse defensive behaviors. Eur. Neuropsychopharmacol. 21 (4), 306–315. 10.1016/j.euroneuro.2010.05.004 20570114 PMC3250220

[B51] Prager E. M. Hadley C. Bergstrom G. H. W. MariaBraga F. M. (2016). The basolateral amygdala γ-aminobutyric acidergic system in health and disease: BLA GABAergic system in health and disease. J. Neurosci. Res. 94 (6), 548–567. 10.1002/jnr.23690 26586374 PMC4837071

[B52] Prut L. Belzung C. (2003). The open field as a paradigm to measure the effects of drugs on anxiety-like behaviors: A review. Eur. J. Pharmacol. 463 (1–3), 3–33. 10.1016/S0014-2999(03)01272-X 12600700

[B53] Ran W. Xue X. (2018). Theranostical application of nanomedicine for treating central nervous system disorders. Sci. China Life Sci. 61 (4), 392–399. 10.1007/s11427-017-9292-7 29675554

[B54] Ren C. Hu X. Zhou Q. (2018). Graphene oxide quantum dots reduce oxidative stress and inhibit neurotoxicity *in vitro* and *in vivo* through catalase-like activity and metabolic regulation. Adv. Sci. 5 (5), 1700595. 10.1002/advs.201700595 PMC5978962 29876205

[B55] Riedel W. J. Klaassen T. Griez E. Honig A. Menheere P. P. Van Praag H. M. (2002). Dissociable hormonal, cognitive and mood responses to neuroendocrine challenge: Evidence for receptor-specific serotonergic dysregulation in depressed mood. Neuropsychopharmacology 26 (3), 358–367. 10.1016/S0893-133X(01)00361-X 11850150

[B56] Risbrough V. B. Stein M. B. (2006). Role of corticotropin releasing factor in anxiety disorders: A translational research perspective. Hormones Behav. 50 (4), 550–561. 10.1016/j.yhbeh.2006.06.019 PMC1884405 16870185

[B57] Ross S. Wu R. S. Wei S. C. Ross G. M. Chang H. T. (2020). The analytical and biomedical applications of carbon dots and their future theranostic potential: A review. J. Food Drug Analysis 28 (4), 677–695. 10.38212/2224-6614.1154 PMC9261808 35696139

[B58] Russo S. J. Nestler E. J. (2013). The brain reward circuitry in mood disorders. Nat. Rev. Neurosci. 14 (9), 609–625. 10.1038/nrn3381 23942470 PMC3867253

[B59] Saitoh A. Kimura Y. Suzuki T. Kawai K. Nagase H. Kamei J. (2004). Potential anxiolytic and antidepressant-like activities of SNC80, a selective δ-opioid agonist, in behavioral models in rodents. J. Pharmacol. Sci. 95 (3), 374–380. 10.1254/jphs.fpj04014x 15272214

[B60] Santomauro D. F. AnaHerrera M. M. Shadid J. Zheng P. Ashbaugh C. Pigott D. M. (2021). Global prevalence and burden of depressive and anxiety disorders in 204 Countries and Territories in 2020 due to the COVID-19 pandemic. Lancet 398 (10312), 1700–1712. 10.1016/S0140-6736(21)02143-7 34634250 PMC8500697

[B61] Sarasa S. B. Mahendran R. Muthusamy G. Thankappan B. Femil Selta D. R. Jayaraman A. (2020). A brief review on the non-protein amino acid, gamma-amino butyric acid (GABA): Its production and role in microbes. Curr. Microbiol. 77 (4), 534–544. 10.1007/s00284-019-01839-w 31844936

[B62] Singh I. Arora R. Dhiman H. Pahwa R. (2018). Carbon quantum dots: Synthesis, characterization and biomedical applications. Turkish J. Pharm. Sci. 15 (2), 219–230. 10.4274/tjps.63497 PMC7228020 32454664

[B63] Tafet G. E. Idoyaga-Vargas V. P. Abulafia D. P. Calandria J. M. Roffman S. S. Chiovetta A. (2001). Correlation between cortisol level and serotonin uptake in patients with chronic stress and depression. Cognitive, Affect. Behav. Neurosci. 1 (4), 388–393. 10.3758/CABN.1.4.388 12467090

[B64] Tafet G. E. Nemeroff C. B. (2020). Pharmacological treatment of anxiety disorders: The role of the HPA Axis. Front. Psychiatry 11 443. 10.3389/fpsyt.2020.00443 32499732 PMC7243209

[B65] Wang Y. F. Liu L. Xue X. Liang X. J. (2017). Nanoparticle-based drug delivery systems: What can they really do *in vivo*? F1000Research 6 681. 10.12688/f1000research.9690.1 28620465 PMC5461891

[B66] Wee N. J. v. d. Frederieke van Veen J. Stevens H. Irenevan Vliet M. Petervan Rijk P. Westenberg H. G. (2008). Increased serotonin and dopamine transporter binding in psychotropic medication–naïve patients with generalized social anxiety disorder shown by ^123^ I-β-(4-Iodophenyl)-Tropane SPECT. J. Nucl. Med. 49 (5), 757–763. 10.2967/jnumed.107.045518 18413401

[B67] Wei X. Li L. Liu J. Yu L. Li H. Cheng F. (2019). Green synthesis of fluorescent carbon dots from gynostemma for bioimaging and antioxidant in zebrafish. ACS Appl. Mater. Interfaces 11 (10), 9832–9840. 10.1021/acsami.9b00074 30758177

[B68] Wirth A. Holst K. Ponimaskin E. (2017). How serotonin receptors regulate morphogenic signalling in neurons. Prog. Neurobiol. 151 35–56. 10.1016/j.pneurobio.2016.03.007 27013076

[B69] Xiao S. Zhou D. Luan P. Gu B. Feng L. Fan S. (2016). Graphene quantum dots conjugated neuroprotective peptide improve learning and memory capability. Biomaterials 106 98–110. 10.1016/j.biomaterials.2016.08.021 27552320

[B70] Xie W. Meng X. Zhai Y. Zhou P. Ye T. Wang Z. (2018). Panax notoginseng saponins: A review of its mechanisms of antidepressant or anxiolytic effects and network analysis on phytochemistry and pharmacology. Molecules 23 (4), 940. 10.3390/molecules23040940 29673237 PMC6017639

[B71] Xu Y. Lian Y. Li J. Zhang Y. Liu Y. Wang X. (2022). KangPiLao decoction modulates cognitive and emotional disorders in rats with central fatigue through the GABA/Glu pathway. Front. Pharmacol. 13 939169. 10.3389/fphar.2022.939169 36120289 PMC9478895

[B72] Xue X. Yang J. Y. Yi H. Wang L. R. Liu P. Li-Sha Y. (2016). Aggregated single-walled carbon nanotubes attenuate the behavioural and neurochemical effects of methamphetamine in mice. Nat. Nanotechnol. 11 (7), 613–620. 10.1038/nnano.2016.23 26974957 PMC5535299

[B73] Yamamoto K. I. Shinba T. Yoshii M. (2014). Psychiatric symptoms of noradrenergic dysfunction: A pathophysiological view. Psychiatry Clin. Neurosci. 68 (1), 1–20. 10.1111/pcn.12126 24372896

[B74] Yu W. Wang L. Yang L. Li Y. J. Wang M. Qiu C. (2020). Activation of LXRβ signaling in the amygdala confers anxiolytic effects through rebalancing excitatory and inhibitory neurotransmission upon acute stress. Neurotherapeutics 17 (3), 1253–1270. 10.1007/s13311-020-00857-y 32297184 PMC7609627

[B75] Zangrossi H. Graeff F. G. (2014). Serotonin in anxiety and panic: Contributions of the elevated T-maze. Neurosci. Biobehav. Rev. 46 397–406. 10.1016/j.neubiorev.2014.03.007 24657635

[B76] Zhang N. Zhang L. Feng L. Yao L. (2018). Cananga odorata essential oil reverses the anxiety induced by 1-(3-chlorophenyl) piperazine through regulating the MAPK pathway and serotonin system in mice. J. Ethnopharmacol. 219 23–30. 10.1016/j.jep.2018.03.013 29545208

[B77] Zhang Y. Wang S. Lu F. Zhang M. Kong H. Cheng J. (2021). The neuroprotective effect of pretreatment with carbon dots from crinis carbonisatus (carbonized human hair) against cerebral ischemia reperfusion injury. J. Nanobiotechnology 19 (1), 257. 10.1186/s12951-021-00908-2 34454522 PMC8399708

[B78] Zhou X. D. Shi D. D. Zhang Z. J. (2019). Ameliorative effects of Radix rehmanniae extract on the anxiety- and depression-like symptoms in ovariectomized mice: A behavioral and molecular study. Phytomedicine 63 153012. 10.1016/j.phymed.2019.153012 31301535

[B79] Zirak N. Shafiee M. Soltani G. Mirzaei M. Sahebkar A. (2019). *Hypericum perforatum* in the treatment of psychiatric and neurodegenerative disorders: Current evidence and potential mechanisms of action. J. Cell. Physiology 234 (6), 8496–8508. 10.1002/jcp.27781 30461013

[B80] Zweifel L. S. Fadok J. P. Argilli E. Garelick M. G. Jones G. L. TavisDickerson M. K. (2011). Activation of dopamine neurons is critical for aversive conditioning and prevention of generalized anxiety. Nat. Neurosci. 14 (5), 620–626. 10.1038/nn.2808 21499253 PMC3083461

